# Correlates of knowledge of family planning among people living in fishing communities of Lake Victoria, Uganda

**DOI:** 10.1186/s12889-020-09762-7

**Published:** 2020-11-03

**Authors:** Annet Nanvubya, Rhoda K. Wanyenze, Teddy Nakaweesa, Juliet Mpendo, Barbarah Kawoozo, Francis Matovu, Sarah Nabukalu, Geoffrey Omoding, Jed Kaweesi, John Ndugga, Onesmus Kamacooko, Kundai Chinyenze, Matt Price, Jean Pierre Van Geertruyden

**Affiliations:** 1 UVRI-IAVI HIV Vaccine Program, Plot 51-59, Nakiwogo Road, P.O Box 49, Entebbe, Uganda; 2 Global Health Institute, University of Antwerp, Antwerp, Belgium; 3 School of Public Health, Makerere University College of Health Sciences, Kampala, Uganda; 4 Uganda Virus Research Institute, Entebbe, Uganda; 5 IAVI, New York, NY USA; 6 Department of Epidemiology and Biostatistics, University of California at San Francisco, San Francisco, CA USA

**Keywords:** Family planning, Fishing communities, Awareness, Satisfactory knowledge

## Abstract

**Background:**

Knowledge of family planning (FP) is a key determinant of contraceptive use which ultimately plays a role in attainment of good health and in conduct of clinical research. People living in fishing communities (FCs) have limited access to health services including FP and are targeted for future clinical research but their knowledge of FP and its correlates are scantily known. We determined correlates of knowledge of FP among people living in FCs of *L. victoria* in Uganda to inform future FP education programs in FCs.

**Methods:**

We conducted a comparative cross-sectional survey among participants aged 15–49 years from Kigungu and Nsazi. Participants were asked if they were aware of any FP method. All those who responded in the affirmative were further asked to mention what FP methods they had heard of or knew. Those who reported knowledge of at least one FP method were asked a series of questions about FP methods and their side effects. Knowledge was categorized into good or poor knowledge based on their mean total score. Poor knowledge constituted a score below the mean while good knowledge constituted a score of more than or equal to the mean total score. To further explore attitudes and perceptions of FP, ten in-depth interviews and four focus group discussions were conducted.

**Results:**

Of the 1410 screened participants, 94.5% were aware of at least one FP method. Pills and injectable hormonal methods were the most commonly known methods. Slightly over a third (38%) had good knowledge of FP. Correlates of knowledge of FP were; being female (aOR: 1.92 95% CI: 1.39–2.67), residing in Kigungu (aOR: 4.01 95% CI: 2.77–5.81), being married (aOR: 1.59 95% CI: 1.11–2.28) and currently being in a sexual relationship (aOR: 1.75 95% CI: 1.18–2.60). Concerns about safety and effectiveness of some modern FP methods exist. Misconceptions on effects of FP like sterility, cancers and foetal abnormalities were common.

**Conclusion:**

FP awareness among people living in FCs of *L. Victoria* in Uganda is high. However, good knowledge about specific methods tends to be low. Correlates of knowledge of FP include gender, residence, marital status and sexual engagement.

**Supplementary Information:**

The online version contains supplementary material available at 10.1186/s12889-020-09762-7.

## Background

Contraception enables families to achieve their desired number of children and is instrumental in child spacing [1]. However, the number of children a couple may have and their child spacing interval tend to be influenced by their knowledge and eventual use of contraception [2, 3]. Due in part to lack of adequate knowledge of family planning (FP), some Ugandan women start bearing children at an early age and continue giving birth until late ages leading to a high fertility rate of 5.4 births per woman of reproductive age [4, 5]. This poses challenges for safe motherhood and child survival as well as other development programmes aimed at improving the quality of life of the population at large.

In Uganda, the National health policy (NHP) provides an overall framework for the health sector and it guides the thinking and implementation of health services including FP provision. The goal of the NHP II is to attain a good standard of health for all people in Uganda in order to promote healthy and productive lives [6]. In an effort to improve FP uptake, the Ministry of Health (MOH), in collaboration with other partners, developed the Uganda Family Planning Costed Implementation Plan, 2015–2020 (FP-CIP) to provide national guidance for increasing the knowledge of and access to FP methods [7]. A social and behaviour change communication’s strategy was developed to ensure honest, accurate, clear, and consistent FP messaging while targeting various audiences. This strategy was implemented through a mass media campaign using radio, television and print messages to supplement the face to face FP counselling that was done by both health and non-health workers to create awareness and educate masses on FP. To date, the extent to which this strategy has improved FP knowledge in some rural sub-populations where other social services are known to be poor is not known. This study provides the opportunity to know how this plan has benefited the fishing communities.

With regard to HIV, Ugandan women tend to be disproportionately affected compared to men [5]. According to the results of the 2016 Uganda population HIV impact assessment, adult HIV prevalence was higher among women at 7.5% compared to 4.3% among men. These rates are presumed to be even higher in marginalized settings. Integration of HIV prevention services within contraceptive services across the country is critical in the reduction of the spread of HIV/AIDS among reproductive women [8]. Integration of health services offers women who may be stigmatized the opportunity to access many services in one location. This has been observed in Madagascar to increase FP uptake while reducing associated stigma [9]. Integration however, can only be maximized if the users have adequate knowledge of the services.

To ensure good health and wellbeing for people in subpopulations with high HIV infection rates, good access to and use of contraception is important [2]. We note however, that the contraceptive prevalence rate of Uganda is one of the lowest in sub-Saharan Africa [10]. It is only slightly higher than that of Tanzania and is much lower than that of other East African countries like Rwanda and Kenya [11, 12]. We are also cognisant of the fact that there are social, structural and economic barriers in FP service coverage that still exist in Uganda and other East African countries which tend to be worse in some sub-populations [13].

Knowledge of FP has been associated with contraceptive use in different settings [14, 15]. In Uganda, knowledge of FP varies across regions and it is unknown for some sub-populations [16]. To ensure that people can use contraception when desired, it would be desirable that all the gaps that may hinder universal assess of FP such as poor, inaccurate and inadequate knowledge are bridged. People living in fishing communities (FCs) in Uganda make a great contribution to food security, foreign exchange & local government revenue and they contribute close to 30% of the country’s gross domestic product [17] . But due to the geographical locations of FCs, the residents have limited access to reproductive health and other social services [18–20]. Moreover, these FCs have many commercial sex workers, high rates of transactional sex (i.e. sex for money, fish, or other goods) and elevated levels of alcohol consumption [21–23], factors that necessitate continuous use of contraception. Unfortunately, their contraceptive use has been reported to be low [24, 25]. Given their life styles, there remains a need to improve their fertility support services in order to increase their contraceptive use.

Due to the prevailing socio-economic challenges in their healthcare systems, there exists inequities in healthcare provision especially in sub-Saharan Africa [26]. Sometime back, the Pathfinder International and other partners started a project called Health of People and the Environment in the Lake Victoria Basin (HoPE-LVB) in two districts in Uganda and two counties in Kenya [27]. This project was implemented with the aim of promoting a complete status of well-being of individuals. In an attempt to promote fairness in access of healthcare services in rural sub-Saharan Africa, the project recommended that FP initiatives should function while focusing on the context where they operate. Since fishing communities in Uganda are hard-to-reach areas with low literacy levels, it is worthwhile to ensure good and adequate knowledge of FP among people living in FCs if use is to be optimized as has been reported by other studies conducted in similar settings [28–30]. This study established knowledge of FP and its correlates among people living in FCs of Lake Victoria in Uganda. It also explored attitudes, perceptions and reasons that influence contraceptive method preferences with the aim of informing future FP education programs for these communities.

## Methods

### Study population and setting

The study targeted a community wide resident population from Kigungu (mainland) and Nsazi (island) FCs. These two communities were selected from the 8 FCs of the Fisher Folk Community Cohort (FFC) of the Uganda Virus Research Institute-International AIDS vaccine Initiative at Entebbe [31]. They were purposively selected because they were the largest among the 8 communities of the FFC but also because they are among the large communities of the Lake Victoria basin. Because of their high HIV infection rates, they represent study populations being targeted for future HIV vaccine research.

Kigungu is a landing site along the shores of Lake Victoria, approximately 45 min from Entebbe, which is a major town where the international airport is located. It is a rural community with a population of approximately 30,000 people [32]. Many residents live in non-permanent housing structures made of wood [18]. There is one government health centre III facility and a few private clinics where medical services are accessed. A health centre III facility is known to include a labour ward and a few outpatient services among which include free HIV counselling and testing. These facilities also offer male condoms, pills and Injectaplan®/ Depo-Provera® for FP. Community based non-governmental organizations provide intermittent HIV prevention and FP outreach services. A few of the medical services offered include information dissemination, treatment of minor illnesses, distribution of condoms and provision of hormonal FP services. Referrals to healthcare facilities are made for more comprehensive services. Less frequently, mobile HIV counselling and testing, reproductive health services and male circumcision services are offered in the community during health facility outreaches from non-governmental organizations. Most of the characteristics of Kigungu described are similar to the context of other mainland FCs in Uganda, including its rural setting and distance from health care services [20, 33], its highly mobile population [34–36], and the presence of alcohol establishments and commercial sex workers [37] with an average number of 7 children per woman/household [18].

Nsazi Island lies on 7 mile^2^ of land and is one of the Islands on Lake Victoria with a total population that oscillates between 5000 and 8000 due to fish seasonality. There is a lot of business and trade for the men and transactions are often done in cash, spending on alcohol selling and commercial sex which is common [18, 31] . Other common occupations include food vending, bar waiting and commercial sex in a less-formal manner as restaurant maids or assistants in brothels. A few residents are involved in livestock rearing and farming at a subsistence level. Given the remoteness of this fishing community, a government health Centre II facility and private clinics provide access to health services. These facilities are usually manned by unqualified personnel.

### Study design

We conducted a comparative cross-sectional survey between February and November 2017 in the two study villages.

### Selection of participants in the cross-sectional survey

The sample size for the study was obtained from 1786 households on a household list that had been previously generated during census of the FCs [31]. From this list, 1452 eligible households were selected. Individuals who were interviewed for baseline knowledge assessment, were got from the 1452 households. From each eligible household, either the man or woman who was present at the time of interview was a potential participant. In instances where the woman or man were eligible and present, they would agree on who was interviewed. Eligible participants included those aged 15 to 49 years, those willing to participate and those who were resident in these communities for at least 6 months at the time the study. We excluded participants who were not willing to consent for the study or those who were not available for the study duration. Ultimately, a total of 1410 participants were assessed for knowledge of FP.

### Methods of data collection

#### Cross-sectional survey

Prior to commencement of the study, the study team were trained on the study and how to use the study questionnaires. The questionnaires used in the study were specifically developed for the study. Pretesting of the questionnaires was conducted to check the suitability of various aspects of the questionnaires such as the translation, skip actions and clarifying questions. Modifications were done prior to the actual data collection. The study was then presented to community leaders and thereafter to other members in both communities by the research team. Participants were invited to study clinics based in their communities where more study information was provided and study procedures conducted. Written informed consent was obtained from each participant prior to conducting any study related procedures. A well-trained and experienced team of 5 interviewers collected the data. Data were collected on social demographic characteristics, FP methods and other reproductive health aspects using semi-structured questionnaires that were anonymized (refer to Socio-demographic and Family planning questionnaires). Participants were asked if they had heard of or knew any FP method. Those who knew of at least one FP method were then asked to list, unprompted, which methods they knew. Those who reported knowledge of at least one FP method were asked a series of questions about those FP methods that they knew and their side effects (refer to Knowledge Assessment questionnaire). They were also asked to mention any sources of FP information that they knew. Participants were asked what they perceived as the ideal number of children for a couple and the ideal birth spacing interval. Participants were also asked if they were currently using any FP method. Those who were using FP were asked to state the duration for using that method and the source of the method they were using. They were asked if it was very easy, easy, not easy, or not easy at all for them to get FP methods and why they were currently using a particular method. In addition they were asked if they had children and how many children they had. Those who were not using FP were asked to give reasons why.

#### Measures

Our outcome of interest was good knowledge of FP. All participants who responded in the affirmative when they were asked if they were aware of any FP method were reported as being aware of FP. Knowledge level was assessed through a series of questions on different FP methods and their side effects. The following FP methods constituted questions on knowledge assessment; pills, injectable hormonal methods, implants, emergency contraceptive pills, intra-uterine device, vasectomy, tubal ligation, condoms, spermicides, diaphragm, withdraw, breast feeding (lactation amenorrhea),calendar, moon beads, periodic abstinence, foam/jelly, herbs and dermal patch. To assess knowledge of FP, a total of 64 questions were asked in regard to which options were known, eligibility criteria, mechanisms and duration of action, routes of administration, adverse effects of methods known, how these adverse effects can be managed and what needs to be done in case of a missed dose or if a replacement is required, other benefits of FP besides contraception and FP use in the context of HIV. Questions that were correctly answered were scored 1 while those that were not correctly answered or where the participant said they didn’t know the answer were scored 0. Knowledge level was categorized into good or poor knowledge based on the mean total score. Poor knowledge constituted a score below the mean while good knowledge constituted a score of more than or equal to the mean total score. The perceived ideal number of children was recorded as ≤4 for those who said their ideal number was 4 or fewer children or > 4 for those who said their ideal number was more than 4 children. The perceived ideal birth spacing interval was recorded as < 2 for those who said their ideal birth spacing interval was fewer than 2 years or ≥ 2 for those who said their ideal birth spacing interval was 2 or more years. Participants’ use or non-use of FP was set as a binary outcome variable.

#### Qualitative data collection

We explored general FP understanding, attitudes and perceptions of FP and preferred FP methods through ten in-depth interviews (IDIs) and four focus group discussions (FGDs) which were stratified by age and sex. The interviews and discussions were conducted using study guides which study guides included semi-structured, open and close-ended questions that elicited information about participants’ knowledge of FP, sociocultural beliefs and practices, perceptions of and attitudes to FP use. Reasons that influenced preference, and choices for methods were also elicited. All discussions and interviews were conducted until saturation was reached by experienced facilitators in either English or Luganda. Saturation was determined when participants in the FGD or an IDI participant had no further questions to ask and opinions or comments to make when all topics on the study guides had been addressed. Two research assistants who were fluent in both English and Luganda took detailed notes during discussions and interviews. The discussions and interviews were also audio recorded. Qualitative Data were transcribed in the English language. In order to maintain confidentiality, the discussions and interviews were conducted in a private environment and the transcripts did not bear participant names. The final transcripts were stored securely on laptops and external drives that were password-protected.

#### Selection of participants in the qualitative component

The participants of the FGDs and IDIs were selected based on their professions, their roles in the study communities and their perceived knowledge of the subject matter by community members. Purposive sampling was used to recruit as varied a sample as possible in order to gather a wide range of responses. The FGDs were conducted in different age categories and gender (there was one female and one male FGD of those aged 15–17 years, one FGD of males aged 25–49 years and one FGD of females aged 25–49 years). Each FGD comprised of 8–11 participants. The IDI participants were either local leaders, health, religious or youth representatives that were recommended by community gate keepers who were either political, social or cultural heads in the two communities. To identify participants, we worked in collaboration with a resident community mobilizer and the Beach Management Unit (BMU) at the landing site. The BMU is an elected organized group of local leaders at any fishing community that represents the interests of the community.

#### Data management and analysis

With regard to quantitative data, data generated from questionnaires were coded and edited before entry into Microsoft Access. Data were reviewed for completeness and accuracy before analysis was done. Data were double entered in Microsoft Access then later cleaned and exported to STATA 15.0 (StataCorp, College Station, TX, USA) for analysis. We resolved discrepancies by checking the source documents. We obtained frequencies and percentages of demographic data stratified by village of residence. We used logistic regression to determine factors associated with good knowledge of FP. Factors for which the association attained statistical significance on log likelihood ratio test (LRT) of *p* < 0.10 were selected for the multivariable logistic regression model at unadjusted analysis. We retained factors in the final multivariable logistics regression model if their inclusion did not make the fit of the model significantly worse at the 5% level on a likelihood ratio test (LRT). Models were adjusted to eliminate potential confounders in reference from findings of other FP studies [14, 28, 38, 39].

For the qualitative data, the primary author initially read all the transcripts and field notes. She listed key statements, ideas, opinions and attitudes expressed which were reflecting the original domains of the interview guide. This was helpful in identifying emerging themes from the data. A preliminary set of sub-themes based on repetitive patterns were identified. A coding framework or schedule was developed based on priori themes in the interview guide. The coding was discussed and verified by the first, second and last author. Data from each transcript were coded by the first author and was further examined by the second and last author who discussed in detail areas of consensus and disagreements. Data were analysed using a thematic approach [40], with support of NVivo-12 qualitative software.

## Results

### Socio-demographic profile of participants in the survey

A total of 1410 individuals participated in the study, majority (1143; 81%) of whom were from Kigungu (Table 1). More than two thirds (911; 65%) of the participants were aged 15–29 years. Slightly more than a third (514; 36%) were engaging in fishing or a fishing related activity. Most (590; 42%) of them were Catholics while half (706; 50%) of them had attained only up to primary level of education with very few (106; 8%) in both villages reaching the tertiary education level. Most (1043; 74%) of the participants had stayed in the community for more than 12 months. Majority (1157; 82%) of the participants reported being in a sexual relationship even though just over a half (810; 58%) of the participants were married. In both villages, those who reported having multiple sexual partners in the past 12 months were fewer (534; 38%) than those who reported not having multiple sexual partners (876; 62%) in the past 12 months. Nearly all participants said the ideal number of children for a couple was four or fewer children (1134; 80%) and the ideal spacing interval was 2 or more years (136; 97%). The reported ideal number of children and spacing interval was congruent to what was actual in more than two thirds of the participants.

**Table 1:** Socio-demographic profile of participants in the cross sectional survey stratified by village

Characteristic	Total (***N*** = 1410) n (col %)	Kigungu (***n*** = 1143) n (col %)	Nsazi (***n*** = 267) n (col%)	Chi-Square ***P***-value
Mean Age (SD)	27.5(7.2)	27.1(7.1)	29.1(7.4)	< 0.001
Median Age (IQR)	26(22–32)	25(21–32)	28(23–34)	< 0.001
Age group (Years)				0.011
15–29	911(65)	759(66)	152(57)	
30–39	397(28)	308(27)	89(33)	
40+	102(7)	76(7)	26(10)	
**Tribe**				0.111
Muganda	631(45)	512(45)	119(44)	
Munyankole	129(9)	114(10)	15(6)	
Musoga	96(7)	71(6)	25(9)	
Mukiga	31(2)	24(2)	7(3)	
Munyarwanda	123(9)	103(9)	20(8)	
Other^a^	400(28)	319(28)	81(30)	
**Occupation**				< 0.001
Farming	33(2)	28(2)	5(2)	
Fishing/Fishing related	514(36)	380(33)	134(50)	
Trade/business	275(20)	234(20)	41(15)	
House wife	124(9)	92(8)	32(12)	
Other^b^	464(33)	409(36)	55(21)	
**Religion**				0.001
Catholic	590(42)	478(42)	112(42)	
Protestant/Anglican	339(24)	265(23)	74(27)	
Muslim	238(17)	182(16)	56(21)	
Other^c^	243(17)	218(19)	25(9)	
**Highest Education level**				0.001
No formal education	82(6)	70(6)	12(6)	
Primary level	706(50)	554(48)	152(57)	
Secondary level	516(37)	419(37)	97(36)	
Tertiary level	106(8)	100(9)	6(2)	
**Sex**				0.057
Male	697(49)	579(51)	118(44)	
Female	713(51)	564(49)	149(56)	
**Marital status**				< 0.001
Single/ Never Married	343(15.7)	301(26.3)	42(15.7)	
Not married	810(57.5)	652(57.0)	158(59.2)	
Divorced/Separated/Widowed	257(18.2)	190(16.6)	67(25.1)	
**Duration of stay**				< 0.001
Months	367(26)	266(23)	101(38)	
Years	1043(74)	877(77)	166(62)	
**Are you currently in a sexual relationship?**				0.008
Yes	1157(82)	923(81)	234(88)	
No	253(18)	220(19)	33(12)	
**Having multiple sexual partners in past 12 months**				0.026
No(< 2 partners)	876(62)	726(64)	150(56)	
Yes(≥ 2 partners)	534(38)	417(36)	117(44)	
**Ideal Number of children for a couple**				0.007
Said ≤4 Children	1134(80)	935(82)	199(75)	
Said > 4 Children	276(20)	208(18)	68(25)	
**Ideal birth spacing interval**				0.284
Said < 2 years	48(3)	41(4)	7(3)	
Said ≥2 years	1362(97)	1102(96)	260(97)	

^a^ (Mugisu, Itesot, Non-Ugandan), ^b^ (Sex worker, Teacher, Security personnel and others), ^c^ (Pentecostal/ Born again, Traditional African, No religion

### Knowledge of family planning methods

Almost all (1333; 94.5%) the participants were aware or knew at least one FP method (Table 2). Pills (1027; 77%), injectable hormonal methods (1004; 75%), implants (776; 58%) condoms (607; 52%) and IUDs (636; 48%) were the most commonly known methods in both villages. Knowledge of specific methods tended to be slightly higher among participants in Kigungu than those in Nsazi. Knowledge of permanent methods (vasectomy and bilateral tubal ligation) was low in both villages and it ranged between 3.2 and 9.6%. Knowledge of natural or traditional methods (periodic abstinence, calendar, breast-feeding rhythm/withdrawal, moon beads) was also low in both villages ranging between 0.4 and 13.8%. Some methods such as emergency pills and spermicides were not known in Nsazi.

**Table 2:** Knowledge of family planning methods

**Methods**	**Number (N = 1410)**	**Kigungu (N = 1143)**	**Nsazi (N = 267)**
Knowledge of any FP method	1333 (94.5%)	1086 (95%)	247 (92.5%)
**Knowledge of specific methods**^**a**^	**Number (*N*** **= 1333)** **(n, %)**	**Number (*N*** **= 1086)** **(n, %)**	**Number (*N*** **= 247)** **(n, %)**
Pills	1027(77.0)	843 (77.6)	184(74.5)
Injectable hormonal methods	1004(75.3)	812 (74.8)	192(77.7)
Implants	776(58.2)	621(57.1)	155(62.8)
Condoms	697(52.3)	623(57.4)	74(30)
IUD	636(47.7)	503(46.3)	133(53.8)
Rhythm	155(11.6)	150(13.8)	5(2)
Vasectomy	112(8.4)	104(9.6)	8(3.2)
Tubal Ligation	97(7.3)	89(8.2)	8(3.2)
Periodic Abstinence	78(5.9)	74(6.8)	4(1.6)
Calendar	54(4.1)	52(4.8)	2(0.8)
Breast feeding/LAM	40(3.0)	39(3.6)	1(0.4)
Emergency Pill	34(2.6)	34(3.1)	0(0)
Spermicide	31(2.3)	31(2.9)	0(0)
Herbs	28(2.1)	24(2.2)	4(1.6)
Moon beads	22(1.7)	18(1.7)	4(1.6)
Diaphragm	5(0.4)	4(0.4)	1(0.4)
Foam	3(0.2)	3(0.3)	0(0)
other	1(0.1)	1(0.1)	0(0)

^a^Knowledge of each method was assessed independently out of 100%

### Family planning knowledge classification

**Fig. 1:**
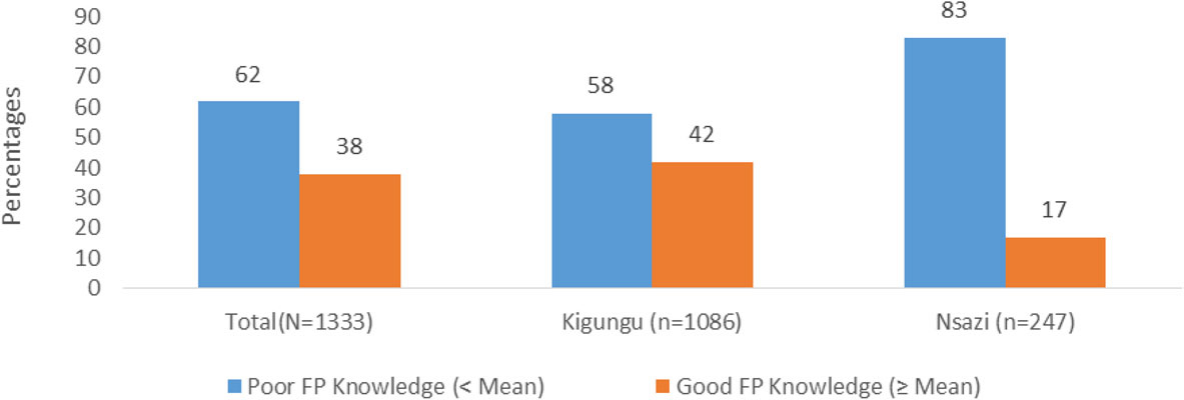
Family planning knowledge classification

When participants who knew at least one FP method (1333;94.5%) were further assessed on knowledge of FP methods and their side effects, the minimum score was 1/64 (2%) and maximum score was 40/64(63%) with a mean score of11/64 (17%). Of the 1333 participants who knew at least one FP method, slightly above a third (502; 38%) had good knowledge on FP methods and their side effects. Majority (205; 83%) of the participants in Nsazi had poor knowledge (Fig. 1).

### Correlates of knowledge of FP in FCs

At unadjusted analysis, statistically significant correlates of good knowledge of FP included; sex, type of employment, level of education, village of residence, marital status, duration of stay in the village and currently being in a sexual relationship. After adjustment, factors that remained statistically significantly associated with good knowledge of FP were sex, village of residence, marital status and currently being in a sexual relationship. Good knowledge of FP was significantly higher among females than males (aOR: 1.92 95% CI: 1.39–2.67). It was also significantly higher among residents of Kigungu than Nsazi (aOR: 4.01 95% CI: 2.77–5.81), among those who were married compared to those who were single (Never married) (aOR: 1.59 95% CI: 1.11–2.28) and among those currently in a sexual relationship compared to those who were not (aOR: 1.75 95% CI: 1.18–2.60). Slightly over a third (502, 35.6%) reporting using FP with the most commonly used methods being condoms, pills and injectable hormones. Good FP knowledge was found to be significantly associated with use of family planning (Table 3).

**Table 3:** Correlates of Knowledge of Family planning in fishing communities of *L. victoria*, Uganda

Characteristic	Total (***N*** = 1333) n (col %)	Good knowledge (***N*** = 502) n (col %)	uOR 95%CI	***p***-value	aOR 95%CI	***P***-value
**Age group (Years)**				0.323		
15–29	861(64.6)	329(65.5)	Ref			
30–39	380(28.5)	145(28.9)	0.99(0.79–1.28)		1.04(0.79–1.39)	0.766
40+	92(6.9)	28(5.6)	0.71(0.44–1.13)		0.83(0.50–1.38)	0.467
**Sex**				< 0.001		
Male	633(47.5)	198(39.4)	Ref			
Female	700(52.5)	304(60.6)	1.69(1.35–2.11)		**1.92(1.39–2.67)**	**< 0.001**
**Tribe**				0.407		
Muganda	603(45.2)	229(45.6)	Ref			
Munyankole	121(9.1)	46(9.2)	1.0(0.67–1.50)			
Musoga	91(7.0)	42(8.4)	1.40(0.89–2.18)			
Mukiga	29(2.2)	13(2.6)	1.33(0.64–2.81)			
Munyarwanda	116(8.7)	38(7.6)	0.80(0.52–1.21)			
Other^a^	373(28.0)	134(26.7)	0.92(0.70–1.20)			
**Occupation**				0.004		
Farming	34(2.6)	12(2.4)	Ref			
Fishing/Fishing related	471(35.3)	147(29.3)	0.83(0.40–1.73)		1.06(0.49–2.30)	0.875
Trade/business	435(32.6)	179(35.7)	1.28(0.62–2.66)		1.09(0.50–2.35)	0.83
House wife	123(9.2)	45(9.0)	1.06(0.48–2.34)		0.64(0.27–1.50)	0.306
Other^b^	270(20.3)	119(23.7)	1.44(0.69–3.04)		1.28(0.58–2.79)	0.542
**Highest Education level**				0.067		
No formal education	80(6.0)	27(5.4)	Ref			
Primary level	652(48.9)	231(46.0)	1.08(0.66–1.76)		1.20(0.72–2.00)	0.488
Secondary level	498(37.4)	210(41.8)	1.43(0.87–2.35)		1.63(0.96–2.77)	0.071
Tertiary level	103(7.7)	34(6.8)	0.97(0.52–1.80)		1.12(0.57–2.22)	0.725
**Religion**				0.584		
Catholic	560(42.0)	202(40.2)	Ref			
Protestant/Anglican	324(24.3)	126(25.1)	1.13(0.85–1.50)			
Muslim	217(16.3)	89(17.7)	1.23(0.89–1.70)			
Other^c^	232(17.4)	85(16.9)	1.02(0.75–1.41)			
**Residence**				< 0.001		
Nsazi	267(20)	42(8.4)	Ref			**< 0.001**
Kigungu	1143(85.7)	460(91.6)	3.89(2.52–5.11)		**4.01(2.77–5.81)**	
**Marital status**				< 0.001		
Single (Never Married)	311(23.0)	92(18.3)	Ref			
Married	777(58.3)	327(65.1)	1.73(1.30–2.29)		**1.59(1.11–2.28)**	**< 0.001**
Divorced/Separated/Widowed	245(18.4)	83(16.5)	1.22(0.85–1.75)		1.37(0.90–2.08)	0.141
**Duration of stay**				0.009		
< 12 Months	343(25.7)	109(21.7)	Ref			
≥ 12 Months	990(74.3)	393(78.3)	1.41(1.09(1.83)		1.27(0.96–1.68)	0.096
**Are you currently in a sexual relationship?**				< 0.001		
No	1105(82.9)	60(11.9)	Ref			
Yes	228(17.1	442(88.1)	1.87(1.36–2.57)		**1.75(1.18–2.60)**	**0.005**
**Having multiple sexual partners in past 12 months**				0.062		
No(< 2 partners)	834(62.6)	330(65.7)	Ref			
Yes(≥ 2 partners)	499(37.4)	172(34.3)	0.80(0.64–1.01)		1.00(0.76–1.32)	0.98
**Ideal number of children**				0.436		
Said ≤4 Children	1077(80.8)	411(81.9)	Ref			
Said > 4 Children	256(19.2)	91(18.1)	0.89(0.67–1.19)			

^a^ (Mugisu, Itesot, Non-Ugandan), ^b^ (Sex worker, Teacher, Security personnel and others), ^c^ (Pentecostal/ Born again, Traditional African, No religion) (*uOR* Unadjusted odds ratio, *aOR* Adjusted odds ratio; *CI* Confidence Interval)

### Sources of family planning information

When participants were asked to mention the sources of FP information that they knew existed in their village, nearly all (1212; 91.1%) indicated governmental hospitals, more than half (870; 65.3%) mentioned private hospitals or clinics while less than a third (336; 25.2%) mentioned non-governmental organizations (NGOs). Only a few in both villages mentioned Traditional Birth Attendants (TBAs) as sources of FP information. Kigungu (44; 4.1%), Nsazi (3; 1.2%) respectively (Table 4). Other sources included; pharmacy or drug shops, family planning clinics, drug or medicine vendors, ordinary retail shops and friends among others.

**Table 4:** Sources of family planning information known^a^

Source	Total (***N*** = 1333) (n, %)	Kigungu (***N*** = 1086) (n, %)	Nsazi (***N*** = 247) (n, %)
Government hospital	1215(91.1)	1009(92.9)	206(83.4)
Private hospital/clinic	870(65.3)	727(66.9)	143(57.9)
Non-governmental Organizations (NGOs)	336(25.2)	301(27.7)	35(14.2)
Pharmacy/drug shop	140(10.5)	131(12.1)	9(3.6)
Family planning clinics	131(9.8)	115(10.6)	16(6.5)
Drug/Medicine vendors	54(4.1)	53(4.9)	1(0.4)
Ordinary shop	48(3.6)	39(8.3)	9(3.6)
Traditional Birth Attendants (TBAs)	47(3.5)	44(4.1)	3(1.2)
Others	32(2.4)	32(2.9)	0(0)

^a^Each source was assessed independently out of 100%

### Findings from the qualitative aspect of the study

Each FGD comprised of 8–11 members. IDIs were conducted with significant members of the community including; a community advisory board member, religious leader, political/ local council leader, commercial sex worker, TBA, Village Health Team member (VHT) also referred to as community health workers (CHWs) and some other recognized community leaders. The FGDs and IDIs comprised of 47 participants (Table 5). FGDs lasted between 65 and 103 min while the IDIs lasted between 37 and 75 min. We identified four themes relevant to knowledge of FP which included: 1) General community understanding and awareness of FP, 2) Beliefs and Attitudes towards FP, 3) Known sources of information on FP with their related challenges and 4) perceived reasons for or choices of preferred methods.

**Table 5:** Description of FGD and IDI participants

	FGD Description	Number in Group	Duration in Minutes	Fishing Community
1.	Females	11	90	Nsazi
	Aged 25–49 years			
2.	Males	8	103	Kigungu
	Aged 25-49 years			
3.	Females	9	65	Kigungu
	Aged 15-17 years			
4.	Males	9	85	Nsazi
	Aged 15-17 years			
	IDI Description			
1.	Community Advisory Board Member		37	Kigungu
2.	Religious leader		56	Kigungu
3.	Political/Local Council leader		61	Nsazi
4.	Medical Personnel		70	Kigungu
5.	Representative from High risk group (Fisherman)		63	Nsazi
6.	Traditional Birth Attendant		45	Nsazi
7.	Female Peer leader aged 25 years		72	Kigungu
8.	Female Sex worker aged 17 years		50	Nsazi
9.	Male Peer leader aged 27 years		75	Kigungu
10.	Male Youth leader aged 19 years		55	Nsazi

### General community understanding and awareness of FP

The first theme which emerged revealed that the community members generally understood the concept of FP and that they were all aware of at least one FP method. The Methods that were mentioned included pills, injectable methods such as Depo-Provera® or injectaplan®, condoms, implants, intra-uterine device, vasectomy, bilateral tubal ligation, withdrawal, calendar method, breast feeding and abstinence. Although the awareness of FP methods was high, participants didn’t seem to know much about how and for how long most methods work. While some appreciated that FP was for both limiting the number of births and allowing a good spacing interval between births, there were others who thought FP may affect future fertility or even induce permanent sterility. A respondent in an in-depth interview said, “*The understanding of family planning in this community is that it is used to completely stop one from getting children and yet it should really be for spacing births. Majority think that when you use family planning you stop giving birth because your eggs get damaged.*”(Female, 48 years).

Like what was observed in the survey, most of the community members were mostly aware of modern FP methods like pills, injectable hormonal methods, implants, intra-uterine devices and condoms. There were participants who knew about both modern and natural or traditional methods of FP. They however mentioned the complexity of using the natural or traditional methods which they said were not reliable. Many of the participants knew that condoms can prevent both pregnancy and sexually transmitted infections (STIs) and commented that condoms were popular. They however said that using condoms consistently was difficult especially for the men who think that condoms reduce sexual satisfaction. A few thought that condoms are the only FP method for men. There are others who said condoms were difficult to use in a married setting resulting in mistrust and misunderstandings in the home. Some expressed concerns about limited knowledge on condom use among the youth saying that the youth may be stigmatised and shy away from getting the required FP knowledge before engaging in sexual activities.

Some participants didn’t know about the female condom and the few who knew about it neither knew how it works nor where it can be accessed if one wanted to use it.

Some of the knowledge community members had about FP was inaccurate. Although many have heard about injectable methods for females, there are those who said that they heard that men too have injectable hormonal methods of FP. Some said that vasectomy can make a man fail to get an erection or release sexual fluids.

Like the female condom, some modern FP methods were either not known or not mentioned at all by the focus group or interview participants such as the diaphragm, spermicides, dermal patch and others. Some participants mentioned ineffective methods such as use of herbs and remains of an umbilical cord to prevent conception. One IDI participant who is a TBA said, *“….I also know some herbs that one can use if they don’t want to use those other family planning methods I have listed*”. (Female, 48 years) The use of herbs was attributed to low levels of education by some participants who doubted their effectiveness. The use of remains of an umbilical cord was cited by some as a medically unproven FP method.

### Beliefs and attitudes towards FP

It was noted that people had divergent beliefs and attitudes towards FP. Although some were supportive of FP, negative and incorrect beliefs still exist concerning effects of FP on women’s reproductive health and health in general. We observed that some participants believed that FP can lead to sterility, cancer of the uterus, abnormal uterine masses and foetal abnormalities or disability. A participant from a focus group of males aged 16–17 years said,” *people fear to use a coil [IUD] because they think it can cause cancer or lead to barrenness”*. Because the menstrual cycle changes in some women who are using FP, some participants believe that women who miss their periods, a side effect to some methods of FP, end up getting uterine masses.

Side effects of some FP methods were pointed out such as weight gain or loss, menstrual irregularities or excessive prolonged bleeding, loss of sexual desire and reduced vaginal secretions. Some said that prolonged bleeding, loss of sexual desire and reduced vaginal secretions interfere with sexual activities which later result into family disputes. A participant in a focus group of males aged 15–17 years said,” *Family planning is a long term issue which requires one to decide on what to do during the long periods of ‘no sex’ depending on the methods of choice used; some family planning methods make women lose their sexual desire. Some men cannot do without sex for a long time and that creates problems in the family.*” There are still some who report that some FP methods cause congenital abnormalities or abnormal features in those children born to mothers using FP. Some do not trust information on FP because they think health workers promote FP for monetary gains.

Most of the participants think that FP should be used by women and youth. They attribute this to the shift in gender roles where women in FCs bear the burden of fending for the homes and children. The youth are thought to have very little information on FP and yet they are reported to be mobile and promiscuous. A participant in an in-depth interview said,” *The men here tend to have many women. So if you get many children, you as the woman will suffer because you will bear the burden of feeding them, treating them and taking them to school. Our husbands these days neglect their roles of being heads of families. The women do everything. Because women are left to do everything, they end up engaging in other sexual relationships to get money*.” (Female, 40 years).

Another participant said,” *Women are the ones who should use family planning because women these days have responsibilities like looking for food to feed the children, taking the children for treatment when they fall sick, buying clothes and paying school fees*”. (Female, 17 years) Others said that because of their vulnerability FP should be a woman’s responsibility.

There are some community members who believed that FP was for educated people and yet they thought there were few educated people in FCs.

Men’s awareness of FP was thought to be low compared to that of the women and some report shame in attending FP sessions. One participant in an in-depth interview said,” *It is only a small number of men who have attended family planning sensitisation meetings.*” *The men feel ashamed to go with their wives to family planning sessions, they know it is a ‘woman’s thing’. Because of this, most of the men do not know much about family planning issues.*” (Male, 45 years).

It was observed that both men’s attitudes and their work schedules may hinder them from attending sensitization meetings. Health education campaigns to improve beliefs and attitudes of men towards FP are needed [41].

### Known sources of information on FP and related challenges

Community members get information on FP from various sources, some of which are formal and trusted while others are informal and doubted. The formal sources of information on FP include; health facilities (both governmental and non-governmental), private clinics and media (print, audio and visual). Some of the informal sources include places of worship (churches and mosques), peers, schools, health outreach sessions and village meetings. Regarding sensitization by health workers, the issue of language barrier was one that was mentioned as a challenge to awareness. Because FCs attract job-seekers from across Uganda, there are those who are disadvantaged when they go to health centres where the staff only know English and the village’s local language.

A new trend of using social media as a source of FP knowledge was cited although it was thought to be limited to those with smart phones and computers with internet. One participant from a focus group of males aged 15–17 years said,”…*only updated youth get information about family planning from social media. The reason is not many people are educated enough to use social media or afford it but a few are there*”.

Traditional “Aunties” were also known to provide information on FP even though they were thought to lack formal training. In the Ugandan context, a traditional “Auntie” is a woman (usually advanced in age) who counsels other women on family issues and is entrusted by community members to do so based on her past experience.

VHTs were noted to be another source of information, especially to those who are unable to access health centres due to long distances or stigma. These VHTs, however, were often reported as insufficient sources of FP information. They refer those who require information on long term or permanent methods to big health centres.

### Perceived reasons for preferred methods

In these communities, different factors were reported to inform FP method choice. Some members said that some health facilities or clinics sell specific FP methods and attendees get these methods if they can afford them. A participant from a focus group of female participants aged 15–17 years said, “*If you go to the government health centres, it’s assumed that the medicines or services are free, but at times the health workers demand for some money before the services are provided. So if you have no money, you are denied the service*”.

Others attributed choice of methods to their availability, known side effects of the methods, health worker skills and behaviour, invasiveness of the methods and preference of spouse. A participant from a focus group of female participants aged 15–17 years said, “*Some preferred family planning methods are not readily available at the health centres, and usually the health centres stock methods known to be demanded by most clients, who use the services. A client may want a tubal ligation but health centres cannot do it. They end up referring the client who may not even go where they are referred because they don’t have money for transport.”*

## Discussion

This study assessed correlates of knowledge of FP among people living in FCs of *L. victoria* in Uganda. In this study, we note that in both villages, most of the residents were aged 15–29 years and that most of them had attained only up to primary education level. These being fishing communities, many were engaged in fishing or fishing related activities. We also found that many of them were engaged in sexual relationships. This demographic profile is similar with those from previous studies conducted in the same population [18, 24, 33]. Knowledge of family planning becomes very crucial in a population that is characterized as young with low literacy levels and high sexual activity.

Just like it has been observed elsewhere in the country and in the East African region, we found that nearly all the participants were aware of the concept of FP and that they knew at least one FP method [16, 42–44]. However, when participants were asked if they used any FP method, slightly over one third reported using FP. As countries aim to achieve good health for all, there are global and national efforts to improve knowledge of FP in order to optimise its use. Uganda through the Ministry of Health has supported many sexual and reproductive health campaigns across the country which explains the high awareness of FP [7]. There are also other initiatives that have committed to improvement of FP uptake through creating awareness of FP benefits across the country which also contribute to the high levels of awareness on FP in the FCs [45].

Despite a high awareness of FP, good knowledge of FP was variable and tended to be low. It is crucial for such a population to know how FP methods work, what their actual adverse effects are and to what extent these adverse effects impact health. Misconceptions on effects of FP like inducement of sterility, cancers and foetal abnormalities were common. Accurate and adequate knowledge on the adverse effects of FP can dispel myths and misconceptions about FP and improve its uptake [4]. Use of simple reading materials with information on various FP methods, could help people in FCs to easily make an informed choice when they decide to use FP.

Because of the high infection rates of HIV and other STIs in these communities [46–48], condom use is important for more than just FP. While the male condom was among FP methods that were popularly known, participants only had scanty knowledge about the female condom. Findings also showed that participants lacked knowledge on how the female condom works. To some, it was a new method that they first heard about in the group discussions. If women in FCs are equipped with knowledge on how to use the female condoms and other female specific FP methods, they would be more empowered to manage and control their fertility. Accurate knowledge on the female condom and other female specific FP methods will enable them leverage their choices and actions.

There are misconceptions regarding effectiveness of FP methods that were observed in our study. Some participants think that herbs and remains of an umbilical cord are effective FP methods. This is contrary to what has been found in another study on FP knowledge in Uganda [28]. A systematic review evaluating contraceptive education interventions showed that a range of educational interventions can increase knowledge [49]. People in these FCs may require continuous FP training to equip them with additional knowledge and help dispel some of these misconceptions.

In our study, we observed that participants residing in Kigungu were more likely to have good knowledge of FP compared to those residing in Nsazi. This could be explained by the fact that Kigungu and other mainland communities can easily access health services including FP education services from the general population which may be difficult for participants on Island communities. The mainland communities also tend to have better health services as compared to Island communities.

Being married and current sexual engagement were associated with good knowledge of FP. It is presumed that married people get access to FP information when they go for antenatal care during pregnancy and child birth. It is also likely that those engaging in sexual activity seek for FP information because they want to prevent unintended pregnancies which might explain these findings. Therefore, since most of the participants were found to be engaging in sexual relationships, it may be worthwhile to improve knowledge of FP for everyone including those who are single or never married.

Although some scholars have noted that literacy may impact knowledge of FP in more literate populations [38, 50], our findings didn’t really show that education level was a correlate of knowledge of FP. The majority of participants have a low education status which has been evidenced from other studies in the same population [22, 51]. Regardless of their low literacy levels, the majority of participants said that their ideal number of children was less than 4 children and the ideal birth spacing interval was more than 2 years which tallied with the actual number of children and birth spacing interval for most participants. This shows that a couple’s ideal family size and frequency of births are not ultimately determined by their level of education. But since education level tends to influence comprehension of information, it might be worthwhile to design FP sensitization materials that are easy to understand by the majority. We envisage that visual aids may be useful in enhancing understanding.

We observed that the female gender was statistically significantly associated with good knowledge of FP. Qualitative data also revealed that men in FCs tend to have no interest in FP issues and their knowledge about FP is low perhaps indicating that FP programs are experiencing challenges with targeting men. This is similar to what was observed in another study that explored contraceptive knowledge, perceptions and concerns among men in Uganda [28]. It could also be explained by the fact that females get more sensitization on FP when they go for antenatal care visits during pregnancy.

In another study by Tilahum et.al., it was shown that some men tend to dominate when it comes to deciding on family matters and yet they are reluctant to get information about FP [52]. The lifestyle of most men in FCs is such that majority have multiple sexual partners and have ready access to money from fish sells. They establish homes when they move from one fishing community to another in search for fish. Also culturally, African men tend to desire to have many children as has been observed in our study and in other studies on FP [1] because many children are associated with prestige, masculinity and respect in society [4]. All these make them prone to engaging in sexual relations which sometimes result into unwanted pregnancies. Having good knowledge about FP and knowing when and how to use it is very crucial for all men including those in FCs hence making men an important target group for education campaigns on FP [41].

There is a belief that FP is only for the educated which is contrary to what has been reported in other studies on FP where FP is known to be for all regardless of one’s education status [28, 53]. If some people in FCs believe that FP is only for the educated, this might hinder their use of FP which highlights an important gap in FP messaging that could be filled by a more robust FP education program for this population [49]. On the other hand, the effects of FP such as excessive bleeding, menstrual irregularities, loss of sexual desire and weight changes were reported like in other studies [14, 30, 49] however, the participants had no knowledge of how these can be managed. It is possible that uptake could be improved when people are equipped with knowledge of possible FP effects and how they are managed.

Our study showed that residents of FCs get FP information from various sources which include but are not limited to trained health workers, VHT members, social media, TBAs, posters, school, places of worship, radios including community radios, Television and Traditional aunties (Sengas). Although some of the sources may be authentic with reliable information, some of these sources are very unreliable as has been proven by other studies. The quality and accuracy of information from VHTs, TBAs and traditional aunties is questionable because these are not formally trained to disseminate health information [49].

FCs in Uganda are characterised by their limited health care and other social services [24, 47, 48, 54, 55]. Both Nsazi and Kigungu have one government health facility at health centre II/III level which provide reliable FP information. They have few health workers and even the few were reported not to be skilled enough to provide information on surgical FP methods because they do not offer these services. The non-governmental health facilities or research organizations tend to offer reliable information but haphazardly because of their fixed work schedules. Unlike governmental health facilities that operate for longer hours and almost on a daily basis, the non-governmental facilities operate only on week days for a specified duration which makes it difficult for community members to access health services at any time. Because of a limited number of health facilities in these communities, there is an urgent need for capacity building in form of reproductive health infrastructure and human resource. Government’s continued guidance and support in ensuring a sizeable skilled force in these remotely located settings will be invaluable as the country aims at attainment of SDGs particularly in the FCs.

VHT members and TBAs have been used to bridge the gap in delivery of health services to remote settings. According to World Health Organization (WHO), VHTs or CHWs should be members of the communities where they work, should be selected by the communities and should be answerable to the communities for their activities. They should be supported by the health system but may not necessarily be part of its organization. They tend to have shorter training in regard to healthcare than professional health workers.

In Uganda, a VHT member is a permanent resident of a given community, assigned by government to provide promotive, preventive, limited curative care, rehabilitative, palliative and referral services in relation to maternal, neonatal, child and adolescent health [56]. They help in ensuring good nutrition, creation of awareness and distribution of FP methods ranging from condoms, oral contraceptive pills to injectable contraceptives, reporting of communicable and non-communicable diseases in their community and are held accountable by the ministry of health for the non-performance of these services [56]. They happen to be a powerful health work force that promote and extend the reach of primary and preventative health services closer to the people in their communities. At a small scale, they sensitize and provide medical services to the people. Task shifting has been made possible by the VHTs whereby FP methods including injectable contraceptives are made available even at the lower levels of the health system while relieving the burden at higher healthcare levels. This task shifting helps to solve the human resource crisis and improve access of FP services. Because the VHTs work is voluntary, they tend to be cost effective and affordable. They help in sensitizing masses on FP, manage refills and provide new doses as and when needed. It has been noted however, that there are some challenges in the current VHT system which include their varying levels of training in health service delivery. Because the VHTs work as volunteers, their motivation tends to be affected and some stop serving in that capacity.

TBAs on the other hand, are locally based and mostly old women who deliver mothers outside a formal health care setting. While these are usually not formally trained, their expertise is built on experiences and what they have been trained to do by older TBAs. It is therefore important to recognise that VHTs and TBAs are existing sources of health care in the communities and can improve service delivery through community based approaches. It might be worthwhile to empower them with adequate and accurate knowledge of FP and other services. Since TBAs get in contact with women at delivery, they can educate the pregnant women on spacing of next child or contraception needs. Because of the prevailing inadequate human resource in terms of number and expertise in these communities, VHTs and TBAs should be periodically trained to refresh their knowledge and skills. Motivating them through monetary compensation may be beneficial in maintaining their services. Quality assurance mechanisms of the VHTs and TBAs however, will be needed for better outcomes.

### Strengths and limitations

Due to financial constraints, the study was conducted in two FCs selected for their size and location, suggesting that our data might not be fully generalizable to other communities on the lake or elsewhere. We observed a difference in knowledge across the two communities which could have been attributed to the few participants recruited from the island community. Asking participants if they knew any FP methods and their side effects could have led to participants negatively thinking about the methods hence resulting in biases. To gain greater insight into knowledge of FP methods and their side effects, attitudes and beliefs about FP, we additionally employed qualitative methods.

## Conclusion

From this study, we conclude that FP awareness in FCs is high with a wide range of methods known. However, good knowledge about specific methods is variable and tends to be low. Some of the fisher folk still believe in ineffective methods that are not scientifically proven FP methods. Misconceptions about effectiveness of some methods and side effects still exist. The ideal family size is generally less than 4 children while the ideal birth spacing interval is generally more than 2 years. The correlates of FP knowledge were found to be female gender, residence, sexual engagement and marital status. To improve knowledge of FP in FCs, continuous comprehensive education on FP methods and their effects is needed.

## Supplementary Information

**Additional file 1.** Socio-demographic questionnaire. Socio-demographic characteristics of study participants.

**Additional file 2.** Knowledge Assessment questionnaire. Questions about different family planning methods and other reproductive health aspects with their responses.

**Additional file 3.** Family planning questionnaire. Data on sexual activity, fertility issues and family planning use.

## Data Availability

The datasets used and/or analysed during the current study are available from the corresponding author on reasonable request. A full data set containing the data supporting the study findings in this article can also be obtained from the Program Data Manager, by email to: tnakaweesa@iavi.or.ug or information@iavi.or.ug.

## Abbreviations

AIDS: Acquired Immunodeficiency Syndrome
BMU: Beach Management
CI: Confidence Interval
CSWs: Commercial Sex Workers
FP: Family Planning
FCs: Fishing Communities
FGD: Focus Group Discussion
HIV: Human Immunodeficiency Virus
IAVI: International AIDS Vaccine Initiative
IDI: In-depth interview
NGO: Non-Governmental Organization
STIs: Sexually Transmitted Infections
TBA: Traditional Birth Attendant
US: United States
VHTs: Village Health Team members
WHO: World Health Organization
