# Supplementary material for: Correlates of knowledge of family planning among people living in fishing communities of Lake Victoria, Uganda
Source: BMC Public Health. 2020 Nov 3;20:1642. doi: 10.1186/s12889-020-09762-7 (PMC7607714; doi:10.1186/s12889-020-09762-7)
Supplement: Supplementary file 1 — Additional file 1. Socio-demographic questionnaire. Socio-demographic characteristics of study participants. [file 12889_2020_9762_MOESM1_ESM.doc]

*Please complete this form* ***ONLY*** *at screening visit*

| 1. Sex 1= Male 2= Female |__| [SEX]  2. When were you born?|__|__|/|__|__|__|/|__|__|__|__| [DOB]  dd/mmm/yyyy  2.1 How old are you now? |__|__| **completed years** [AGE]  3. In which country were you born? |__| [NATION]  1= Uganda  2=Other country, specify:_______________________ [OTNATION]  4. Tribal or ethnic group? |__|__| [TRIBE]  01=Muganda  02=Munyankole  03=Musoga  04=Mukiga  05=Munyarwanda  06=Other Ugandan, specify……………………………….[OTUTRIBE]  07=Other non-Ugandan ,specify………………………….[OTNUTRIBE]  99=Don’t know  5. Main source of income |__|__| [JOBNOW]  01=Working in a bar  02=Working in a night club, disco  03=Working in a restaurant  04=Working in a hotel/guesthouse  05=Hair dresser  06=Working in beauty salon  07=Giving massage  08=Food vendor  09=Vendor of clothes  10=Sex work  11=Fishing  12=Other fish related work  13=Farming  14=Other, specify……………………………………[OTJOBNOW]  15=Several of above, specify………………………[SEVJOBNOW]  16=No job  17= Housewife    6. What is your religion? |__|__| [RELIGION]  01=Catholic  02=Church of Uganda-Anglican  03=Muslim  04=Born Again Christian  05=Seventh Day Adventist  06=Hindu  07=Traditional African  08=No religion  09=Other, specify………………………………………….. [OTRELIG]  7a. Which village are you currently living/working in? |__| [VILLAGE]  1= Kigungu 2= Nsazi  7b. Which District are you currently living/working in? |__| [DISTRICT]    1=Wakiso 2=Mukono  8. How long (months) have you lived in this area?|__|[STAREA]  1=D |__|__|  2=M |__|__|  3=Y |__|__|  9. What is the highest formal school education you obtained?  |__|__| [LEVELED]  00=Never went to school  01=Started but did not complete primary level  02=Completed primary level  03=Started but did not complete ordinary secondary level  04=Completed ordinary secondary level  05=Started but did not complete advanced secondary level  06=Completed advanced secondary level  07=Started any tertiary level (university or other) but did not complete  08=Completed any tertiary level (university or other)  99=Don’t know  10. What is your current marital status? |__| [MARSTAT]  1=Married, monogamous  2=Married, polygamous  3=Widowed  4=Separated/Divorced  5=Single (never married) |  |
| --- | --- |

Form Completed by____________________________________________ _____________________

Signature Date Form Completed

Reviewed by ____________________________________________ _____________________

Signature Date Form Reviewed

Entered by ____________________________________________ _____________________

Signature Date Form Entered
