# Supplementary material for: Correlates of knowledge of family planning among people living in fishing communities of Lake Victoria, Uganda
Source: BMC Public Health. 2020 Nov 3;20:1642. doi: 10.1186/s12889-020-09762-7 (PMC7607714; doi:10.1186/s12889-020-09762-7)
Supplement: Supplementary file 2 — Additional file 2. Knowledge Assessment questionnaire. Questions about different family planning methods and other reproductive health aspects with their responses. [file 12889_2020_9762_MOESM2_ESM.docx]

Assessment date: |__|__|/|__|__|__|/|__|__|__|__| (dd/MMM/ yyyy) [ADATE]

Volunteer ID Number: FP01 -|__|__| - |__|__|__|__| [IDNO] Visit Number: ¦__¦__¦ ¦__¦ [VNO]

**Instructions:**

*For each question, give a mark of 1 if answer is correct and 0 for an incorrect answer.*

*At the end, get the percentage score using this formula;*

*(Total marks ÷64 x 100)*

*To complete table below Q.4; anyone who gets percentage score of ≥ 70%=1 (passed) and whoever gets a score of <70%= 2 (not passed)*

**The Pill (COCs)**

Q: What is the main mechanism of action of the pill (COCs)? ¦__¦

A: Works mainly by stopping ovulation

Q: When can a client start to take pills (COCs), without needing to use additional contraceptive protection? ¦__¦

A: Within the 5 first days of the start of her menstrual period.

Q: Can a woman who is 4 months postpartum and breastfeeding start to use the pill (COCs)? ¦__¦

A: No, she must wait until 6 months postpartum.

Q: When can a woman start to take COCs after an abortion? ¦__¦

A: She can start immediately.

Q: What would you advise a client who missed one pill (COC)? ¦__¦

A: She should take the missed pill as soon as she remembers, and continue taking pills as usual, one each day.

Q: In what circumstance does a woman who missed pills have to skip the pill-free week and go straight to the next pack? ¦__¦

A: If she missed 3 or more pills in week 3.

Q: List 3 return signs for COCs. ¦__¦

A: 3 from the following:

- - Severe, constant pain in belly, chest, or legs
  - Very bad headaches
  - Migraine aura (a bright spot in vision before bad headaches).
  - Yellow skin or eyes

Q: Mention 3 common side-effects of pill use (COCs). ¦__¦

A: 3 from the following:

- - Nausea/upset stomach
  - Spotting or bleeding between periods
  - Mild headache
  - Breast tenderness
  - Dizziness
  - Slight weight gain or loss

Q: Can a woman with varicose veins use the pill (COCs)? ¦__¦

A: Yes.

Q: What type of pills would be recommended for a woman who is breastfeeding?

A: The mini-pill (progestogen-only pills). ¦__¦

**Mini-pill (POPs)**

Q: Describe the 2 mechanisms of action of the mini-pill (POPs)

A: 1) Thickens cervical mucus. ¦__¦

2) Can stop ovulation.

Q: True or false: common side-effects of the mini-pill include headaches, tender breasts and dizziness? ¦__¦

A: False: These side-effects are not common.

Q: If a woman is switching from injectables to the mini-pill, when should she start taking the pills? ¦__¦

A: At the time she would have had the repeat injection.

Q: When can a breastfeeding woman start using the mini-pill?

A: From 6 weeks postpartum. ¦__¦

Q: If a non-breastfeeding woman is late taking her mini-pill by 12 hours, what should she do? ¦__¦

A:

- She should take the missed pill as soon as possible.
- She should avoid sexual intercourse or use a condom for the next 2 days, after restarting the pill.

**Long-acting Injectables**

Q: What type of hormones do long-acting injectables contain?

A: Progestogen (NOT estrogen). ¦__¦

Q: What are the 2 most commonly used long-acting injectables, and how often must they be given? ¦__¦

A:

- DMPA, every 3 months.
- NET-EN, every 2 months.

Q: A 45 year old woman who smokes heavily would like to use DMPA. Can she use this method? ¦__¦

A: No: she has 2 or more risk factors for heart disease.

Q: If a woman comes to the clinic on day 10 of the menstrual cycle, but has not had sex for 2 weeks, and wants to start using long-acting injectables, what should the provider do? ¦__¦

A:

1. Give her an injection now.

2. Ask her to avoid sex or use condoms for the next 7 days.

Q: When after childbirth can a breastfeeding woman start using long-acting injectables? ¦__¦

A: From 6 weeks postpartum

Q: Up to how many days BEFORE her “due date” can a woman receive her DMPA injection? ¦__¦

A: She can come up to 2 weeks early for her injection.

Q: A woman comes to the clinic 3 weeks after her repeat injection date for DMPA; she has not had sex for the past month. What should you do? ¦__¦

A:

- - Give her the injection.
  - Advise her to use condoms or avoid sex for the next 7 days.
  - Discuss how she can remember next time.

**Monthly Injectables**

Q: True or false: Monthly injectables contain the same hormones as the combined pill (COCs). ¦__¦

A: True. They contain estrogen and progestogen hormones.

Q: Can a breastfeeding woman who is 3 months postpartum start using CICs?

A: No. She must wait until 6 months postpartum. ¦__¦

Q: Can a woman who is not medically eligible to take the pill (COCs) use the monthly injectable instead? ¦__¦

A: No, she cannot.

Q: True or false: Before giving the injection, you must swab the skin.

A: False. If the client’s skin is visibility dirty, you should wash it. But no need to swab skin. ¦__¦

Q: If a client is 10 days late for a monthly injection (CIC), does she need to use condoms/avoid sex for the next 7 days? ¦__¦

A: Yes. She must do so if she is more than 7 days late.

**Norplant Implants**

Q: Norplant implants are made of how many plastic tubes? ¦__¦

A: 6 plastic tubes.

Q: True or false: Norplant implants contain progestogen and estrogen hormones.

A: False. They contain only progestogen. ¦__¦

Q: Why would a woman need to have her Norplant implants replaced after 4 years?

A: If she weighs more than 80 kg. ¦__¦

Q: A woman has been using pills and wants to switch to using implants. She is in week 3 of the cycle. Can she have the implants inserted now? ¦__¦

A: Yes.

Q: A woman returns to the clinic who has been using implants for the past 5 years. She weighs 75 kg. Should you: ¦__¦

a) Tell her to come back in 2 years to have her implants replaced?

Or b) Advise her to have her implants replaced now.

A: (B) She should have her implants replaced now.

Q: List 2 common side-effects of Norplant implants? ¦__¦

A: 2 from among:

- Light spotting or bleeding between periods
- Irregular bleeding.
- No monthly bleeding (amenorrhoea).

**Emergency Contraception**

Q: A woman comes into the clinic. She had unprotected intercourse 4 days ago. Can she take the emergency contraceptive pill? ¦__¦

A: Yes. She can take ECPs up to 5 days after unprotected sex.

Q: True or false: the emergency IUD is more effective than emergency contraceptive pills. ¦__¦

A: True. The IUD is more effective than the pills

Q: True or false: emergency contraceptive pills work by causing an abortion.

A: False. They do not cause abortion. They work mainly by stopping ovulation.

¦__¦

**Copper-bearing IUD**

Q: How does the copper-bearing IUD work? ¦__¦

A: It works mainly by stopping the sperm and egg from meeting.

Q: True or false: The copper IUD begins to rust in the uterus if not removed after 5 years. ¦__¦

A: False. The IUD does not rust in the body.

Q: A client comes to the clinic and wants to use the IUD. After a pelvic exam you find that she has vaginitis. Can she have the IUD inserted? ¦__¦

A: Yes, she can.

Q: An IUD user returns to the clinic after 1 year. She is pregnant. The strings are visible. What should you do? ¦__¦

A: Recommend IUD removal, but explain risk of miscarriage.

Q: True or false: An IUD user can take aspirin to help reduce bleeding problems.

A: False: She can take ibuprofen or similar medication, but NOT aspirin.

¦__¦

Q: Up to how many days in the menstrual cycle can a woman have the IUD inserted, without the need for extra protection? ¦__¦

A: Up to 12 days

Q: A woman gave birth 24 hours ago. Can she have an IUD inserted now?

A: Yes. She can have it inserted up to 48 hours postpartum, or after 4 weeks.

¦__¦

Q: List 3 return signs for IUD. ¦__¦

A: 3 from among:

1. Missed a period or thinks she might be pregnant.

2. IUD strings have changed length or are missing.

3. Might have an STI or HIV/AIDS.

4. Bad pain in lower abdomen.

**Vasectomy and Sterilization**

Q: True or false: Vasectomy is more effective than female sterilization?

A: True. It is more effective. ¦__¦

Q: For how long must a man use an additional contraceptive method after getting a vasectomy before the procedure becomes effective? ¦__¦

A: For 3 months after the procedure.

Q: For how long should a woman rest after a sterilization procedure?

A: For 2 to 3 days. ¦__¦

**Condoms and vaginal methods**

Q: Which of these lubricants should NOT be used with a male condom?

1. Clean water ¦__¦

2. Baby oil

3. Spermicides

A: (2) Baby oil and all other oil-based lubricants should not be used.

Q: True or false: The male condom is less effective than the female condom.

A: False: The male condom is more effective. ¦__¦

Q: Up to how many hours before intercourse can the female condom be inserted?

A: Up to 8 hours ahead. ¦__¦

Q: Do spermicides help protect against STIs and HIV? ¦__¦

A: No. And women at high risk of HIV should not use them.

Q: When can a woman have a diaphragm fitted after childbirth? ¦__¦

A: She should wait 6 to 12 weeks after childbirth, depending on when the uterus and cervix return to normal size.

**LAM and FAB**

Q: What does the “A” in LAM stand for? ¦__¦

A: Amenorrhoea.

Q: Which of the following is NOT a condition of LAM: ¦__¦

1. Less than 6 months postpartum

2. Baby feeds well

3. Fully or nearly fully breastfeeding.

4. Periods have not returned.

A: (2) Baby feeds well.

Q: A woman has been using LAM. She is 5 months postpartum. She has started to feed her baby other foods. What should you advise her to do? ¦__¦

A: If she wants to stay protected from pregnancy, she should start using another contraceptive method NOW. LAM is no longer effective.

Q: An HIV positive woman, living in an area with no safe water supply, wants to know if she can breastfeed the baby. What should you advise? ¦__¦

A: Advise her that she should breastfeed fully for 6 months, and then stop breastfeeding.

Q: List 2 advantages of fertility awareness-based methods. ¦__¦

A: 2 from among:

- Do not cause any side-effects.

- Can be effective if used correctly.

- Do not need to take any medication.

- Do not need to come back to the clinic regularly.

- Do not need to buy anything.

- Can be used by women who may not be able to use hormonal methods.

Q: True or false: When using the Standard Days Method, a user must abstain from sex or use condoms for 12 days in a row each cycle. ¦__¦

A: True.

**General on contraception**

Q: List the regular hormonal methods of contraception. ¦__¦

A:

- 1. The pill (COCs)
  2. The mini-pill (progestogen-only pills)
  3. Monthly injectables
  4. Long-acting injectables
  5. Implants

Q: Which of these methods is the most effective at preventing pregnancy?

1. IUD ¦__¦

2. Pills

3. Injectables

A: IUD.

Q: List 3 conditions that can be used to rule out pregnancy. ¦__¦

A: 3 from among:

1. Menstrual bleeding started in last 7 days.

2. No sex since last period.

3. Gave birth in the past 4 weeks.

4. Have been fully or nearly fully breastfeeding AND gave birth in the past 6 months AND had no menstrual period since.

5. Had a miscarriage or abortion in the past 7 days.

6. Has been using a reliable method of contraception correctly and consistently.

Q: If a woman develops migraine headaches while using the pill, what should you advise her? ¦__¦

A: She should switch to another method.

Q: What should a pill-user do if she gets bad diarrhoea? ¦__¦

A: She should follow the instructions for missed pills

Q: A woman had implants inserted 5 months ago. She is worried since she has not had a menstrual period in 2 months. She has no signs or symptoms of pregnancy. What should be done? ¦__¦

a) Do a pregnancy test and advise her to have the implants removed; or

b) Advise her that amenorrhoea (no monthly bleeding) is very common with implant use and reassure her that this is not a sign of illness.

A: (B).

**FAMILY PLANNING KNOWLEDGE ASSESSMENT**

| *To assess Family planning knowledge (FPK), a composite score will be calculated based on 5 variables namely - 1. Awareness about FP methods, 2. Correct knowledge about ideal number of children for a couple, 3. Correct knowledge about ideal birth spacing interval, 4. Correct knowledge about FP methods and their side effects and 5. Interval between last 2 children being ≥ 2 years (only for women with more than one child). The final score will be categorized into satisfactory and unsatisfactory based on a cut-off of 80% of the maximum possible score (which will be 4 for women with one child and 5 for women with more than one child.*  *To get the Total score, use this formula;*  *(Total passed ÷4 x 100) or (Total passed÷5 x 100)*  *Final score, Satisfactory=Total score≥ 80%, Unsatisfactory= Total score<80%* |
| --- |

|  | **Score** | |
| --- | --- | --- |
|  | **1=Passed** | **0= Did not pass** |
| 1. ***Awareness about FP methods*** | | |
| Find from FP Questionnaire Q.13 |  |  |
| 1. ***Correct knowledge about ideal number of***   ***children for a couple*** | | |
| Find from FP Checklist Q.2 |  |  |
| 1. ***Correct knowledge about ideal birth***   ***spacing interval*** | | |
| Find from FP Checklist Q.3 |  |  |
| 1. ***Correct knowledge about FP methods***   ***and their side effects*** | | |
| Find from FPK assessment form |  |  |
| 1. ***Interval between last 2 children being***   ***≥ 2 years (for women with more than one child)*** | | |
| Find from FP Questionnaire (Q. 9d.) |  |  |
| **Total Score** |  |  |
| **Final Score** | **1= Satisfactory** | **2= Un satisfactory** |

Form Completed by ____________________________________________ _____________________

Signature Date Form Completed

Reviewed by ____________________________________________ _____________________

Signature Date Form Reviewed

Entered by ____________________________________________ _____________________

Signature Date Form Entered
