# Supplementary material for: Correlates of knowledge of family planning among people living in fishing communities of Lake Victoria, Uganda
Source: BMC Public Health. 2020 Nov 3;20:1642. doi: 10.1186/s12889-020-09762-7 (PMC7607714; doi:10.1186/s12889-020-09762-7)
Supplement: Supplementary file 3 — Additional file 3. Family planning questionnaire. Data on sexual activity, fertility issues and family planning use. [file 12889_2020_9762_MOESM3_ESM.docx]

Family Planning Protocol: **FAMILLY PLANNING QUESTIONNAIRE**

**VOLUNTEER ID**

***Introduction***

***The UVRI-IAVI HIV Vaccine Program located at the Uganda Virus Research Institute, Nakiwogo, Entebbe, has been conducting research for over 10 years. This time we are conducting research on family planning use as we continue to prepare for HIV Vaccine trials in fishing communities along L. Victoria. Results of this study will help in improving family planning use in these communities and will help us assess preparedness for future HIV vaccine trials.***

***I request you to answer a few questions and provide a blood sample. Females will be requested to provide a urine sample. Questions will take 30-45 minutes of your time and your responses will be kept confidential. Please answer the questions honestly.***

Record time the interview started |__|__|:|__|__| HOURS {STARTTIM}

**Identification Information**

**Volunteer ID: FP01 -|__|__| - |__|__|__|__| [**IDNO] Date ____/____/____ {DATE} dd mmm yyyy

**Visit: ¦___¦¦___¦.¦___¦ [VNO]**

1. Sex: Male 1 |__| {SEX}

Female 2

2. Study Site: Kigungu 1 |__| {VILLAGE}

Nsazi 2

3.Do you Stay here or work here?

Stay 1 |__| {RESSTATUS}

Work 2

### QUESTIONS 4-12 ARE FOR FEMALES ONLY, SKIP TO Q.13 FOR MALES

***Madam, the questions we are going to ask you concern pregnancy and child birth. Your responses are important to this research and shall be kept confidential.***

Q.4a. Have you ever been pregnant? Yes 1 |__| {PREGEVER}

No 2 -------------->Q.13

Q.4b. How long ago was your last pregnancy? **(Code completed days, weeks, months or years. Code 88 in remaining boxes. If don’t remember code 97 in all boxes)**

**|__|__|** {LSTPREGDY}

**|__|__|** {LSTPREWK}

**|__|__|** {LSTPREGMON}

**|__|__|** {LSTPREGYR}

Q.4c.How did the last pregnancy end? |__| {PREGEND}

Live birth/baby 1

Miscarriage/abortion 2

IUFD/Stillbirth 3

Other 4

Specify _______________________________ {OTH PREGEND memo}

Q.4d. Did you want your last pregnancy?

Yes 1 |__| {PREGWANT1}

No 2

Q.5a. How many children are still living? |__|__| {CHILDLIV}

Q.5b. Have you had a miscarriage/abortion before? i.e loss of a pregnancy before 7 months? Yes 1 |__| {MISC12MON}

No 2 ‑‑‑‑‑‑----------‑‑->Q.6

DK 7‑‑‑‑‑‑----------‑‑‑‑>Q.6

Q.5 c. How many miscarriages have you had? |__|__| {MISCTOT}

**(Code 97 for don’t know/don’t remember)**

Q.6a. Are you currently pregnant? Yes 1 |__| {PREGNOW1}

No 2----------------->Q.6b

DK 7 ----------------->Q.8a

Q.6b. If not currently pregnant, would you want to become pregnant?

YES, I am trying to conceive 1 |__| {PREGNOW2}

YES, I want to become pregnant 2

I have mixed feelings about becoming pregnant 3

I do not want to become pregnant 4

Q.6c. How long do you want to wait to become pregnant?

Within 2 years 1 |__| {CHILDWAIT1}

>2years 2

Q.7a. If pregnant, approximately how many months is your current pregnancy? |__|__| {PREGDUR}

Q. 7b. At the time you became pregnant, (with this pregnancy or last pregnancy if not currently pregnant) did you want to become pregnant then?

Yes, right time 1 |__| {PREGWANT2}

Yes, but later, (NOT QUITE RIGHT TIME) 2

Not at all (WRONG TIME) 3

Q. 7c. Did you want to have a baby later on or did you not want to have any (more) Children?

Later on 1 |__| {CHILDWANT}

No more 2

Q. 7d. If later how much later did you want to wait? Within 2 years 1 |__| {CHILDWAIT2}

>2years 2

Q.8a The first time you became pregnant were you in school? Yes 1 |__| {PREGSCH)

No 2-----------------> Q.9

Never attended school/NA 8-----------------> Q.9

Q.8b Did you drop out of school because of a pregnancy? Yes 1 |__| {PREGSCHLT}

No 2

Q.9. Have you ever given birth to a live baby (a baby that cried)? Yes 1 |__| {EVRBIRTH}

No 2

Q.9a If Yes, how many? |__|__| {NOBIRTH}

Q.9b Do you have child (ren) with your current sexual partner? Yes 1 |__| {CHILDCUR}

No 2

Q.9c If Yes, how many? |__|__| {NOCHILD}

Q.9d.What is the interval between your last 2 children? <2 years 1 |__| {CHILDINT}

≥ 2 years 2

Q.10 Would you like to have (more) children?

Yes 1----------------->Q.11b |__| {MOREKIDS}

No 2

DK 7 ----------------> Q.13

Q.11a Why don’t you want to have (more) children? **(UNPROMPTED, Circle all that apply)**  Yes No

Children are expensive to look after and bring up (economic reasons) 1 2 {NOMORKID1}

To give the ones I have better living conditions 1 2 {NOMORKID2}

I already have my desired number of children 1 2 {NOMORKID3}

Pregnancy is risky and can affect my health 1 2 {NOMORKID4}

Many children/pregnancies makes a woman grow old faster (cosmetic reasons) 1 2 {NOMORKID5}

Other, Specify ____________________________________ 1 2 {NOMORKID6}

Q.11b. Why would you like to have more children? **(UNPROMPTED, Circle all that apply)**  **Yes No**

My husband/partner wants more 1 2 {MOREKIDRS1}

I am still young 1 2 {MOREKIDRS2}

A few years in marriage 1 2 {MOREKIDRS3}

I want to have at least a boy child 1 2 {MOREKIDRS4}

I want to have at least a girl child 1 2 {MOREKIDRS5}

Each child comes with his/her own blessing 1 2 {MOREKIDRS6}

More children are security for old age 1 2 {MOREKIDRS7}

To replace those who died 1 2 {MOREKIDRS8}

Because religion does not permit use of FP methods 1 2 {MOREKIDRS9}

To compete with my co-wife/co-wives 1 2 {MOREKIDRS10}

Other, specify __________________________________ 1 2 {MOREKIDRS11}

**[IF VOLUNTEER ANSWERED NO TO Q.10, SKIP Q12]**

Q.12. How long would you wish to wait before having another child? |__| {SPACETIME}

Less than 1 year 1

1 to 2 years 2

Greater than 2 years 3

Never again 4

Unsure of timing 5

Not applicable (cannot have a child) 8

| I would like to talk to you about family planning; there are various ways or methods that a couple can use to delay or avoid a pregnancy. |
| --- |

***Some people use various methods to avoid getting pregnant, for different reasons;***

Q.13 Do you know any Family Planning method used to prevent pregnancy? Yes 1 |__| {FPKNOW}

No 2 --------------> Q.16

Q.14 Which Family Planning methods do you know? **(Unprompted, Circle all that apply) Yes No**

Pills 1 2 {FPKNOW1}

Condom 1 2 {FPKNOW2}

Injectable 1 2 {FPKNOW3}

Spermicide 1 2 {FPKNOW4}

Periodic Abstinence 1 2 {FPKNOW5}

Calendar 1 2 {FPKNOW6}

IUD/coil 1 2 {FPKNOW7}

Breast-feeding/Lam 1 2 {FPKNOW8}

Herbs 1 2 {FPKNOW9}

Tubal ligation 1 2 {FPKNOW10}

Vasectomy 1 2 {FPKNOW11}

Implants/Norplant 1 2 {FPKNOW12}

Rhythm/Withdraw method 1 2 {FPKNOW13}

Diaphragm 1 2 {FPKNOW14}

Dermal Patch 1 2 {FPKNOW15}

Emergency Pill 1 2 {FPKNOW16}

Moon beads 1 2 {FPKNOW17}

Foam/Jelly 1 2 {FPKNOW18}

Other 1 2 {FPKNOW19}

If Other, Specify __________________________________

Q.15. Where can someone get family planning methods/services from? **(UNPROMPTED, Circle all that apply)**

Government hospital/clinic 01 |__|__| {FPSOURCE1}

Private hospital/clinic 02 |__|__| {FPSOURCE2}

NGOs 03 |__|__| {FPSOURCE3}

Pharmacy/drug shop 04 |__|__| {FPSOURCE4}

Ordinary shop/weekly markets 05 |__|__| {FPSOURCE5}

Traditional birth attendants 06 |__|__| {FPSOURCE6}

Family planning clinics 07 |__|__| {FPSOURCE7}

Drug/medicine vendors 08 |__|__| {FPSOURCE8}

Other (specify) ____________________ 09 |__|__| {FPSOURCE9}

Do not know 97 |__|__| {FPSOURCE10}

Q.16 A) Are you or your partner(s) currently using the following Family planning methods? **PROMPT**

Yes 1 |__| {FPUSE}

No 2 🡪Q.23

B) **IF USING ASK**: For how long have you and/or your partner used this method consistently without a break? [**Code completed days, weeks, months or years 8 or 88 for other space in duration coded. Leave duration of other methods not currently used blank].**

C) Where did you get this FP method from (what is the source)? **[Get response in list of sources below]**

**A) USING (circle accordingly)** B**) DURATION** C**) SOURCE**

Yes No DK D W MM YY

Pills 1 2 7 {FPUSING1} |__| |__| |__|__| |__|__|{FPDUR1} |__|__| {SOURCEFP1}

Condom 1 2 7 {FPUSING2} |__| |__| |__|__| |__|__|{FPDUR2} |__|__| {SOURCEFP2}

Injectable 1 2 7 {FPUSING3} |__| |__| |__|__| |__|__|{FPDUR3} |__|__| {SOURCEFP3}

Spermicide 1 2 7 {FPUSING4} |__| |__| |__|__| |__|__|{FPDUR4} |__|__|{SOURCEFP4}

Periodic Abstinence 1 2 7 {FPUSING5} |__| |__| |__|__| |__|__|{FPDUR5} |__|__| {SOURCEFP5}

Calendar 1 2 7 {FPUSING6} |__| |__| |__|__| |__|__|{FPDUR6} |__|__|{SOURCEFP6}

IUD/coil 1 2 7 {FPUSING7} |__| |__| |__|__| |__|__|{FPDUR7} |__|__| {SOURCEFP7}

Breast-feeding 1 2 7 {FPUSING8} |__| |__| |__|__| |__|__|{FPDUR8} |__|__| {SOURCEFP8}

Herbs 1 2 7 {FPUSNG9} |__| |__| |__|__| |__|__|{FPDUR9} |__|__| {SOURCEFP9}

Tubal ligation 1 2 7 {FPUSNG10} |__| |__| |__|__| |__|__|{FPDUR10} |__|__| {SOURCEFP10}

Vasectomy 1 2 7 {FPUSNG11} |__| |__| |__|__| |__|__|{FPDUR11} |__|__| {SOURCEFP11}

Implant/Norplant 1 2 7 {FPUSNG12} |__| |__| |__|__| |__|__|{FPDUR12} |__|__| {SOURCEFP12}

Rhythm/Withdrawal 1 2 7 {FPUSING13} |__| |__| |__|__| |__|__| {FPDUR13} |__|__| {SOURCEFP13}

Diaphragm 1 2 7 {FPUSING14} |__| |__| |__|__| |__|__| {FPDUR14} |__|__| {SOURCEFP14}

Dermal Patch 1 2 7 {FPUSING15} |__| |__| |__|__| |__|__| {FPDUR15} |__|__| {SOURCEFP15}

Emergency Pill 1 2 7 {FPUSING16} |__| |__| |__|__| |__|__| {FPDUR16} |__|__| {SOURCEFP16}

Moon Beads 1 2 7 {FPUSING17} |__| |__| |__|__| |__|__| {FPDUR17} |__|__| {SOURCEFP17}

Foam/Jelly 1 2 7 {FPUSING18} |__| |__| |__|__| |__|__| {FPDUR18} |__|__| {SOURCEFP18}

Other 1 2 7 {FPUSING19} |__| |__| |__|__| |__|__| {FPDUR19} |__|__| {SOURCEFP19}

If Other, specify______________________________________________________________ {OTHFPMEMO}

**SOURCE CODES**

Government hospital/clinic 01

Private hospital/clinic 02

NGOs 03

Pharmacy/drug shop 04

Ordinary shop/weekly markets 05

Traditional birth attendants 06

Family planning clinics 07

Drug/medicine vendors 08

Other __________________ 09

**[IF ALL RESPONSES TO Q.16A “USING” ARE “NO” OR ‘‘DK’’ SKIP TO
Q.23]**

Q.17. How effective is the method you are currently using to prevent pregnancy? |__| {FPEFFTV}

Effective 1

Not Effective 2

Q.18. Does your spouse(s)/partner(s) know that you are currently using any family planning method? Yes 1 |__| {PKNOWFP}

No 2

Don’t know 7

Q.19. Is the decision to use the current family planning method mainly your decision, your partners/spouses, or joint? |__| {FPDECIDE}

Mainly mine (respondent) 1

Mainly spouse/ partner 2

Joint decision 3

Other (specify) _____________ 4

Q.20. How often do you discuss using any family planning method(s) with your spouse/partner**? (If not using any Family planning methods skip to Q.30)** Never 1 |__| {FPDISCUS}

Sometimes 2

Often 3

Always 4

Q.21. What costs do you/your partner (spouse) incur when obtaining any family planning method(s)?

**(Multiple responses are acceptable) CIRCLE ACCORDINGLY.**

**Yes No DK**

Transport 1 2 7 {FPCOSTS1}

Buying FP 1 2 7 {FPCOSTS2}

Treating side effects 1 2 7 {FPCOSTS3}

Other 1 2 7 {FPCOSTS4}

Specify _____________________________ {FPCOST memo}

Q.22a. Is it very easy, easy, not easy, or not easy at all for you to get the birth control/family planning

Methods) you’re currently using? **(Prompted)** |__| {EASEFP}

Very easy 1 ------------>Q.22c

Easy 2 ------------>Q.22c

Somehow easy 3

Not easy 4

Not easy at all 5

Q. 22 b. Why is it difficult to get the birth control/family planning method (s) you’re currently using? **[Unprompted, multiple responses applicable]** **Yes No**

Lack of money to buy FP 1 2 {EASEFPRS1}

Lack of money for transport 1 2{EASEFPRS2}

Supplies run out at my source 1 2 {EASEFPRS3}

I have to get permission from husband/partner to go away from home 1 2 {EASEFPRS4}

I do not want my husband/partner to know 1 2 {EASEFPRS5}

I am too busy at home/work to get time to go for FP 1 2 {EASEFPRS6}

Other, 1 2 {EASEFPRS7}

Specify _____________________________________________ {EASEFPRS memo}

Q. 22c. Why are you using the current family planning method?**(Unprompted, multiple responses applicable)**  **Yes No**

Most effective in preventing pregnancy 1 2 {WHYCURFP1}

Convenient (Easy to use) 1 2 {WHYCURFP2}

Easy to get 1 2 {WHYCURFP3}

Affordable 1 2 {WHYCURFP4}

Other reason(s) 1 2 {WHYCURFP5}

Specify ___________________________________ {WHYCURFP memo}

Q. 22d. **“On a scale of 1-5, 1: Very difficult, 5: Not difficult at all”** How difficult is it to access another

Family planning method other than the one you are using?

Very difficult 1 |__| {FPACCESS}

Difficult 2

Somehow difficult 3

Not difficult 4

Not difficult at all 5

### IF VOLUNTEER IS CURRENTLY USING ANY FP METHOD, i.e., AT LEAST ONE RESPONSE IN Q.16A IS YES, SKIP TO Q.24

Q.23. Why are you not using any method of family planning/birth control? **(Unprompted, multiple responses**

**allowed)** **Yes No**

My Spouse/partner disapproved 1 2 {NOFPRS1}

Infrequent/No sex 1 2 {NOFPRS2}

A few years in marriage 1 2 {NOFPRS3}

I want to have children/get pregnant 1 2 {NOFPRS4}

Religion does not permit use of FP methods 1 2 {NOFPRS5}

My culture encourages having more children 1 2 {NOFPRS6}

I cannot afford to buy FP methods (No money) 1 2 {NOFPRS7}

FP methods have side effects 1 2 {NOFPRS8}

Have other health Concerns 1 2 {NOFPRS9}

FP methods are not effective 1 2 {NOFPRS10}

FP is inconvenient to use 1 2 {NOFPRS11}

Method failed/Got Pregnant 1 2 {NOFPRS12}

I do not know where to get FP methods 1 2 {NOFPRS13}

I cannot afford transport costs to where I get FP methods 1 2 {NOFPRS14}

Lack of sexual satisfaction 1 2 {NOFPRS15}

Menstrual Problems 1 2 {NOFPRS16}

Gained Weight 1 2 {NOFPRS17}

Other reason 1 2 {NOFPRS18}

If other, specify ______________________________ {NOFPRS memo}

***Sexual intercourse is one of the modes of transmission for HIV and other Sexually transmitted diseases. I request to ask questions related to sexual intercourse and STI symptoms. Your responses are important to this research and we will keep them confidential.***

### QUESTIONS RELATED TO SEXUAL BEHAVIORS, PRACTICES AND RELATIONSHIPS

Q.24 Are you currently in sexual relationship **(Any kind of relationship even if not legal)**? |__| {CURRMARR}

Yes 1

No 2‑‑‑‑‑‑‑-‑‑‑-‑‑‑‑‑‑‑>Q.26

Q.25 How many sexual partners do you have? |__|__| {POLYMAR}

**[Record actual # wherever possible, code 97 for Do not know]**

Q.26. In the past 12 months (from _____ to ____), has your marital status changed? **If Yes** how?

Separated 1 |__| {CHANGE12PT1}

Divorced 2 |__| {CHANGE12PT2}

Widowed/Partner died 3 |__| {CHANGE12PT3}

Got married 4

No change 5

Q.27 Do you currently have a sexual partner(s) other than your husband/wife? |__| {CURRLTN}

Yes 1

No 2

Q.28 In the past 12 months (from _____ to ____), how many sexual partners have you had, including your husband (wife/wives), casual partner/s and all other people? **[Record actual number, probe for a given number]**

**[Code 92= a few, 93= a lot/many (03+), no response= 99]** |__|__| {SEXP1YR}

**IF 00 (NONE) SKIP TO Q.38**

Q.29 In the past 12 months (from _____ to ____), how many **new** sexual partners have you had? **[A new partner is one you had sex with for the very first time in your life]**

[**Code 92= a few, 93= a lot/many (03+), no response= 99]** |__|__|{NEWP12M}

Q.30 In the past 12 months (from _____ to ____), have you used condoms during sexual intercourse?

|__| {COND12M}

Yes 1

No 2‑‑‑‑‑‑‑‑‑‑‑‑‑‑‑>Q.33

Q.31 In the past 12 months (from _____ to ____), have you consistently used condoms during sexaul intercourse?

Sometimes/inconsistent 1 |__| {CONDFRQ12}

Always 2

DK 7

Q.32. Why did you use condoms with this partner/s? **(Unprompted, multiple responses)**

Yes No

Prevent HIV transmission/acquisition/re-infection 1 2 (CONDHIV)

Prevent STD transmission/acquisition 1 2 (CONDSTD)

For family planning 1 2 (CONDFP)

Just trying it out 1 2 (CONDTRY)

Other specify___________________________ 1 2 (CONDOTH)

DK/DR 1 2 (CONDNR)

Q.33. Why didn’t you use condoms with this partner/s? **(Unprompted, code up to 2 responses)**

**[Code 88 in the 2nd box if one reason is given]**

Did not have any condoms at that time 01 |__|__|{CON12NVR1}

Could not afford their cost (expensive) 02 |__|__|{CON12NVR2}

Not easily available in our community 03

Partner refusal 04

Trusting partner 05

Knew/know our HIV status 06

Knew/know my HIV status 07

For more pleasure 08

Religion does not allow them 09

Wanted to have child/pregancy 10

Other (Specify) _______________________11

Q.34 During the past 3months (from _____ to ____), how many sexual partners have you had, including your husband (wife/wives), casual partner/s and other people you have had sexual intercourse with? |__|__| {SEXP3MON} **[Record actual number, probe for a given number]**

Q.35 During the past 3 months (from _____ to ____), how many **new** sexual partners have you had? **[A new partner is one you had sex with for the very first time in your life]**  |__|__|{NEWPAT3M}

Q.36 During the previous 3 months (from _____ to ____), have you used condoms during sexual intercourse?

Yes 1 |__| {COND3M}

No 2‑‑‑‑‑‑‑‑‑‑‑‑‑‑‑>Q.38

Q.37 During the previous 3 months (from _____ to ____), have you consistently used condoms during sexaul intercourse?

Sometimes/inconsistent 1 |__| {CONDFRQ3}

Always 2

DK 7

**This marks the end of the questions. Thank you very much for your responses and time.**

Record time the interview ended |__|__|:|__|__| HOURS {ENDTIM}

Completed by ____________________________________________ _____________________

Signature Date Form Completed

Reviewed by ____________________________________________ _____________________

Signature Date Form Reviewed

Entered by ____________________________________________ _____________________

Signature Date Form Entered
